# Evolutionary Engineering of an Iron-Resistant *Saccharomyces cerevisiae* Mutant and Its Physiological and Molecular Characterization

**DOI:** 10.3390/microorganisms8010043

**Published:** 2019-12-24

**Authors:** Berrak Gülçin Balaban, Ülkü Yılmaz, Ceren Alkım, Alican Topaloğlu, Halil İbrahim Kısakesen, Can Holyavkin, Zeynep Petek Çakar

**Affiliations:** 1 Department of Molecular Biology and Genetics, Faculty of Science & Letters, Istanbul Technical University, Maslak, Istanbul 34469, Turkey; berrakgb@gmail.com (B.G.B.); lkyilmaz@yahoo.com (Ü.Y.); alkim.ceren@gmail.com (C.A.); topaloglual@gmail.com (A.T.); kisakesenhi@gmail.com (H.İ.K.); holyavkin@gmail.com (C.H.); 2 Dr. Orhan Öcalgiray Molecular Biology, Biotechnology and Genetics Research Center (ITU-MOBGAM), Istanbul Technical University, Maslak, Istanbul 34469, Turkey

**Keywords:** oxidative stress, evolutionary engineering, stress resistance, *Saccharomyces cerevisiae*, transition metals, iron stress, *PHO84*, adaptive laboratory evolution

## Abstract

Iron plays an essential role in all organisms and is involved in the structure of many biomolecules. It also regulates the Fenton reaction where highly reactive hydroxyl radicals occur. Iron is also important for microbial biodiversity, health and nutrition. Excessive iron levels can cause oxidative damage in cells. *Saccharomyces cerevisiae* evolved mechanisms to regulate its iron levels. To study the iron stress resistance in *S. cerevisiae*, evolutionary engineering was employed. The evolved iron stress-resistant mutant “*M8FE*” was analysed physiologically, transcriptomically and by whole genome re-sequencing. *M8FE* showed cross-resistance to other transition metals: cobalt, chromium and nickel and seemed to cope with the iron stress by both avoidance and sequestration strategies. *PHO84*, encoding the high-affinity phosphate transporter, was the most down-regulated gene in the mutant, and may be crucial in iron-resistance. *M8FE* had upregulated many oxidative stress response, reserve carbohydrate metabolism and mitophagy genes, while ribosome biogenesis genes were downregulated. As a possible result of the induced oxidative stress response genes, lower intracellular oxidation levels were observed. *M8FE* also had high trehalose and glycerol production levels. Genome re-sequencing analyses revealed several mutations associated with diverse cellular and metabolic processes, like cell division, phosphate-mediated signalling, cell wall integrity and multidrug transporters.

## 1. Introduction

After aluminium, iron is one of the most commonly found transition metals in the Earth’s crust. In neutral environments, as well as in biological systems, iron is most often found in its Fe^2+^ (ferrous) and Fe^3+^ (ferric) oxidation states. Most forms of iron found in the environment are insoluble. This property of iron prevents biological systems from accessing this micronutrient. Salt forms are more soluble than other forms. Since the ferrous ion is much more soluble than the ferric ion in the 10^−2^ M range, it is more toxic than the ferric ion. Ferrous iron is more bioavailable than ferric iron and it is favourable for oxidation to the more stable “ferric” form, which gains iron redox potential [1,2,3].

The redox potential range of Fe^3+^/Fe^2+^ can be changed between −0.5 V and +0.6 V depending on the physiological conditions and interactions with coordinating ligands. This redox range and reversible electron transfer potential make iron a crucial element in biological systems. Iron plays an important role in numerous reactions, such as oxygen transport (hemoglobin and myoglobin), electron transfer (cytochromes, catalases, ferredoxins) and DNA synthesis (ribonucleotide reductases) [4,5].

While acknowledging the iron dependency of organisms, excess iron can, however, be toxic. Iron can generate highly reactive hydroxyl radicals via the Haber–Weiss–Fenton reactions. It is known that most of the hydrogen peroxide-related oxidative damage occurs with hydroxyl radicals produced by this reaction chain (see Equation (1)) [2]. The ROS (reactive oxygen species) which occur from this reaction can damage proteins, lipids (lipid peroxidation) and DNA (base modifications) [6,7].

Fe^2+^ + O_2_ → Fe^3+^ + O_2_^−●^

2O_2_^−●^ + 2H^+^ → H_2_O_2_ + O_2_ (1)

Fe^2+^ + H_2_O_2_ → OH^●^ + OH^−^ + Fe^3+^

Considering these properties of iron, it is critical that iron levels in an organism should be maintained at the base level while any excesses should be reduced.

Iron deficiency causes anaemia; however, excess iron may lead to kidney and liver damage (hemochromatosis). In addition, iron overload causes neurodegenerative disorders like Alzheimer’s and Parkinson’s disease. Additionally, some iron compounds are also suspected to be carcinogens [8].

Iron transport systems enable organisms to reach iron in a more controlled manner. Iron transport proteins, such as transferrin, maintain high levels of iron serum concentrations while protecting against toxicity of iron [3]. Thus, iron transport systems help overcome the low solubility problem of iron and prevent against toxicity [9].

Iron is also important for microbial biodiversity. The effect of metals on biofilm formation and biofilm resistance to toxic metals in terms of a time-dependent tolerance have been recently reviewed [10]. Very recently, a consensus statement on the key role and global importance of microorganisms in climate change biology has been published, where it was also mentioned that the ocean warming can alleviate the iron limitation of nitrogen-fixing bacteria, with critical potential effects on the new nitrogen supplied to food webs [11]. Considering the health and nutritional effects of iron, attempts were also made to prepare iron-enriched baker’s yeast in recovery from dietary iron deficiency [12], or to investigate iron-related potential changes in the gut microbiota, such as characterization of the gut (*Gallus gallus*) microbiota upon consumption of an iron biofortified feed diet [13] and studying the effects of a decrease in iron bioavailability to human gut microbiome on the reduction of the growth of potentially pathogenic gut bacteria [14].

In order to keep iron levels balanced, organisms have evolved several mechanisms. In the basic eukaryotic model organism, *Saccharomyces cerevisiae*, iron is maintained by the help of two major iron uptake mechanisms: a high-affinity iron transport system and a low-affinity iron transport system [15]. Apart from these uptake mechanisms, *S. cerevisiae* copes with metal toxicity with the help of a sequestration strategy which involves intracellular chelating or compartmentalization into vacuoles [16].

*S. cerevisiae* is a great model organism to represent basic eukaryotes. The *S. cerevisiae* genome is compact and it is easier to manipulate and identify a gene in *S. cerevisiae* than in the human genome [17,18]. The homologous sequences, which usually show homologous functions, can also help reveal some unknown functions of biomolecules. For example, the genes that are responsible for transition metal homeostasis are highly conserved from yeasts to humans [8]. Smf1p, Smf2p and Smf3p are yeast membrane proteins responsible for the uptake of divalent metal ions (nickel, zinc, copper, cobalt and cadmium). The Nramp2 protein (natural resistance-associated macrophage protein), also called the Dct1 (Dmt1) protein, is a homologue of Smf1p and Smf2p (33–36% identity in amino acid sequence) and it is responsible for metal ion transport in mammals [19]. The transmembrane protein Fre1p of *S. cerevisiae* is also homologous of the “Gp91” (Phox) protein of the NADPH oxidase complex (cytochrome b558) of human phagocytic cells [5]. Many examples can be given for the sequence homology of yeast cells to mammalian cells. 

To gain insight into the iron stress resistance mechanism and understand the response of the basic eukaryote *S. cerevisiae*, evolutionary engineering, an inverse metabolic engineering strategy, has been applied in this study. Evolutionary engineering involves repeating cycles of mutations in the aim of obtaining the desired phenotype. Firstly, the reference strain was randomly mutagenized with the chemical mutagen ethyl methanesulfonate (EMS) and the iron stress was then applied systematically and continuously throughout the batch selection. Mutants selected from the final population were investigated. Eventually, one individual mutant with superior resistance properties was selected for further detailed analysis. Evolutionary engineering has been successfully used in our previous studies that involved a variety of stress types [20,21,22,23,24,25,26,27]. Using this strategy, an iron-resistant mutant has been obtained in the present study. The physiological, transcriptomic and genome re-sequencing properties of the iron-resistant mutant have been characterized. The transcriptome data of the mutant has attracted attention to genes other than the most widely-known iron homeostasis genes and genome re-sequencing data supported many of the findings.

## 2. Materials and Methods

### 2.1. Strain, Media, Cultivation Conditions, Stress Factors

The *S. cerevisiae strain* CEN.PK113-7D (*MATa*, *MAL2-8^c^*, *SUC2*) was kindly provided by Prof. Dr. Jean Marie François and Dr. Laurent Benbadis (University of Toulouse, France). Yeast minimal medium (YMM) containing 2.0% (w/v) glucose (Sigma-Aldrich, Hamburg, Germany), 0.67% (*w*/*v*) yeast nitrogen base) with 5.0 g/L ammonium sulphate and without amino acids (Becton, Dickinson and Company, Sparks, MD, USA) and 2.0% agar (Neogen Corporation, Lansing, MI, USA) for solid media were used for cultivations. Cultivations were performed at 30 °C, 150 rpm in an orbital shaker (Sartorius Certomat Göttingen, Germany), using 500 mL–2 L Erlenmeyer flasks filled with YMM to 20% of the flask volume. Optical density (OD_600_) values were measured using a Shimadzu UV-1700 (Shimadzu, Tokyo, Japan) spectrophotometer. Two mL suspensions in 30% (*v*/*v*) glycerol (MP Biomedicals, Solon, OH, USA) were used for preparing the −80 °C stock cultures.

### 2.2. Evolutionary Engineering Protocol

The reference strain CEN.PK113-7D, named as *905*, was exposed to the chemical mutagen; ethyl methanesulfonate (Sigma-Aldrich, Hamburg, Germany) to obtain a randomly mutagenized initial culture named as *906*, with increased genetic diversity, as described previously [28]. The mutagen allowed 10% of the initial culture to survive. Thus, this genetically diverse population was used as the initial culture in the selection experiments for iron-resistant mutants. The minimum inhibitory concentration (MIC) of iron that caused 50% growth reduction was determined previously (Supplemental Figure S1), and 5 mM FeCl_2_ (Sigma-Aldrich, Hamburg, Germany), was applied as the initial, mild iron stress level of selection. For this purpose, *906* cultivated in YMM containing 5 mM FeCl_2_ was named as the first passage and upon cultivation for 24 h, it was transferred to fresh medium containing a slightly higher FeCl_2_ stress level, as the next passage. For all passages of selection, a culture in YMM and without FeCl_2_ was grown in parallel as a control culture. In each successive passage, iron concentration was gradually increased and it finally reached up to 30 mM FeCl_2_ at the 15th passage. Survival was determined by dividing the OD_600_ value of the culture or passage grown with FeCl_2_ to that of the same culture grown under control conditions [21].

### 2.3. Cross-Resistance Determination

#### 2.3.1. Most Probable Number (MPN) Method

MPN method estimates viable cell numbers in culture with 95% confidence [29]. To quantify stress resistance levels, MPN assay was performed in 96-well plates at 30 °C. Twenty microlitres of cultures with OD_600_ of ~1.0 were inoculated to five parallel wells containing 180 µL YMM with and without (control) the stress factor, and then serially diluted by 10-fold in each of the eight following rows. MPN scores were determined upon 48 and 72 h of incubation. Each score refers to the “number of organisms per unit volume” of the original sample and was calculated from the tables indicated (Lindquist) [30]. The survival rates of the cultures were calculated by taking the ratio of the number of cells/mL under stress condition to that of those under control conditions.

#### 2.3.2. Spot Assays

Four OD_600_ unit of cells at an OD_600_ of ~1.0 were serially diluted up to 10^−5^ dilution, and 3 µL were spotted on YMM-agar medium containing a variety of stress factors at a final concentration of 0.3 mM CuSO_4_ (Sigma-Aldrich, Hamburg, Germany), 20 mM MnSO_4_ (Sigma-Aldrich, Hamburg, Germany), 25 mM MnCl_2_ (Merck, Darmstadt, Germany), 10 mM ZnCl_2_ (Sigma-Aldrich, Hamburg, Germany), 2mM CrCl_3_ (Acros organics, Geel, Belgium) , 25 mM FeCl_2_ (Sigma-Aldrich, Hamburg, Germany), 0.3 mM NiCl_2_ (Merck, Darmstadt, Germany), 2 mM CoCl_2_ (Sigma-Aldrich, Hamburg, Germany), 50 mM (NH_4_)_2_Fe(SO_4_)_2_ (Merck, Darmstadt, Germany), 250 µM bathophenanthroline sulphonate (BPS) (Alfa Aesar Karlsruhe, Germany), 0.5 mM H_2_O_2_ (Merck, Darmstadt, Germany) and 2 mL/L phenylethanol (Sigma-Aldrich, Hamburg, Germany). The plates were observed and photographed after incubation at 30 °C for 48 h.

### 2.4. Physiological Analysis

#### 2.4.1. Determination of Cellular Iron and Cobalt Contents

Cellular iron contents were determined using a flame atomic absorption spectrometer (F-AAS) (AA 280 FS, Varian, Victoria, Australia). The wavelength and slit width values were 248.3 nm and 0.2 nm, respectively. Air-acetylene flame was used. For measurements, 2 mM (NH_4_)_2_Fe(SO_4_)_2_ which corresponds to 0.11 mg Fe atom/mL was applied as the iron stress factor for 90 min at 30 °C and 150 rpm. The stress factor was then removed and the cells were dried at 80 °C. The measured cell dry weights for each triplicate sample were used to calculate mg Fe atoms/g cell dry weight values. Five millilitres of 10 M HNO_3_ (Sigma-Aldrich, Hamburg, Germany) was added to each 5 mL sample and incubated at 80 °C for cell lysis. After lysis, 10 mL distilled water was added to have a three-fold dilution prior to the F-AAS measurements**.** Iron contents were then calculated according to known standards**.** Cellular cobalt contents were also determined using the same procedure, with slight modifications: 2.5 mM CoCl_2_ was used as the cobalt stress factor for 90 min at 30 °C. The wavelength and slit width values were 240.7 nm and 0.2 nm, respectively.

#### 2.4.2. Extracellular Metabolite (Glucose, Ethanol, Acetate, Glycerol) Determination

Growth physiological experiments in batch cultures and extracellular metabolite analyses were performed as described previously [23]. Cultures were grown both in the presence and absence of 15 mM (NH_4_)_2_Fe(SO_4_)_2_ as the iron stress factor, which corresponds to 0.83 mg Fe atom/mL.

#### 2.4.3. Reserve Carbohydrate (Glycogen, Trehalose) Determination

A total of 25 OD_600_ units of cells were harvested from each culture. One-millilitre aliquots were collected to calculate cell dry weight values at five different time points. The intracellular trehalose and glycogen content of the samples were determined in the presence and absence of 15 mM (NH_4_)_2_Fe(SO_4_)_2_ as the iron stress factor, as described previously [23]. α,α-Trehalose glucohydrolase ≥1.0 units/mg protein (Cat. No. T8778, Sigma-Aldrich, Hamburg, Germany) was used for the degradation of trehalose to glucose. Amyloglucosidase, 3500U (Cat. No. 11202367001, Sigma-Aldrich, Hamburg, Germany) was used for the degradation of glycogen to glucose.

#### 2.4.4. Oxidative Level Determination

##### Determination of the Intracellular ROS Amounts by Fluorescent Intensity Measurements

A previously described protocol has been followed, with slight modifications [31]. Briefly, pre-cultures were cultivated until an OD_600_ of 1–1.2. Approximately, 1.4 × 10^8^ cells were harvested and pre-incubated for 10 min at 30 °C. 2′,7′-Dichlorodihydrofluorescein diacetate (DCFH-DA) (Sigma-Aldrich, Hamburg, Germany) was applied for 30 min. After probe penetration and washing, the pellet was re-suspended in 1000 µL buffer. Vortexing cells with glass beads was repeated 10 times. The supernatant of the lysate was diluted by 10- or 20-fold to detect the fluorescence. Iron stress was applied before dye treatment to prevent 2’,7’-dichlorofluorescin (DCFH_2_) oxidation by the stress factor.

##### Lipid Peroxidation Assay

Pre-cultures were cultivated until an OD_600_ of 1–1.2. Iron stress was applied throughout the cultivation. Approximately 1.4 × 10^8^ cells were harvested, washed twice with dH_2_O and re-suspended in 1 mL dH_2_O. As the starting material, 500 µL of each sample were used. A previously described protocol has been followed [32].

#### 2.4.5. Lyticase Susceptibility Assay

Lyticase susceptibility test was adapted from a previously described method [33]. Overnight cultures of the reference strain (*905*) and the iron-resistant mutant (*M8FE*) strain were cultivated in 50 mL YMM in 250 mL-shake flasks by initiating from 0.2 OD_600_ (approximately 2.8 × 10^6^ cells mL^−1^), both in the presence and absence (control) of 15 mM (NH_4_)_2_(Fe)(SO_4_)_2_ stress and grown at 30 °C, 150 rpm until stationary phase. The cultures were then harvested by centrifugation at 10,000 *g* for 10 min and an OD_600_ of 0.9 mL^−1^ cells were resuspended in 10 mL of 10 mM Tris/HCl buffer (pH 7.4) including 40 mM β-mercaptoethanol (Merck, Hohenbrunn, Germany). After the samples were incubated at 25 °C for 30 min, 2U/mL^−1^ lyticase (Sigma-Aldrich, St. Louis, MO, USA) was added into each sample and incubated at 30 °C, 150 rpm. The lyticase susceptibility of the cells was monitored by measuring the decrease in OD_600_. The measured OD_600_ values were divided by the initial OD_600_ value and the ratio was multiplied by 100 to calculate the lyticase resistance. This test was performed as three biological repeats.

### 2.5. Transcriptomic Analysis

#### 2.5.1. One Colour DNA-Microarray Analysis

For transcriptomic analysis, cultures were grown in the presence and absence of 15 mM (NH_4_)_2_Fe(SO_4_)_2_ as the iron stress factor. A previously described protocol has been followed, with slight modifications [23]. Briefly, RNA samples with a RIN value of >9 were chosen for microarray analysis and the obtained signals were normalized using the quantile technique. Signals detected by 100% in each probe set have been filtered. Probe sets were then filtered according to the coefficient of variations with a cut-off value of 50%. Significantly changed expressions were acquired by moderated *t*-test with a *p*-value of <0.05. Entities were determined with a fold change cut-off value of 2.0, with *905* as the control condition. For significance analysis, the corrected *p*-value cut-off was chosen as 0.05. Benjamini Hochberg correction was performed [34]. *p*-value computation was chosen as asymptotic (Agilent Technologies, Santa Clara, CA, USA). The complete microarray data are available at GEO repository under accession number GSE61317.

#### 2.5.2. Microarray Validation by Quantitative RT-PCR (qRT-PCR) Analysis

RNAs isolated for microarray analysis stored at −80 °C were used for the qRT-PCR validation of the microarray results. Initial RNA concentrations were adjusted to 1 µg/µL for cDNA synthesis. Transcriptor High Fidelity cDNA Synthesis Kit (Cat. No. 05 081 955 001, Roche, Mannheim, Germany) was used for qRT-PCR and the kit protocol was followed. A total of 50 pmol/µL anchored-oligo(dT)18 primer was used for cDNA synthesis. A LightCycler 480 SYBR Green I Master kit (Cat. No. 04 707 516 001, Roche, Mannheim, Germany) was used for qRT-PCR analysis (Roche LightCycler 480 II, Indianapolis, IN, USA). Gene expression results were normalized to that of the *ACT1* gene (β-actin, reference gene). The primer sets for selected genes with varying transcript abundance were designed by using the Primer-3-Plus assistance programme [35]. The reference strain (*905,* control) sample was used as the standard. The error rate of each run was smaller than 0.2 and efficiencies were between 1.8 and 2.2. The calculations were performed according to comparative CT method [36]. Average 2^−∆*C*t^ values were calculated from CP values obtained from three individual runs. Relative expression levels were calculated by normalizing 2^−∆∆*C*t^ values to that of the reference strain (*905,* control). Cycling conditions were as follows: denaturation for 10 min at 95 °C, followed by 45 cycles of 10 s at 95 °C, 18 s at 56 °C, 20 s at 72 °C, and one cycle of melting for 5 s at 95 °C, 1 min at 65 °C. The reaction was completed with denaturation at 40 °C for 10 s. For each run, melting curve analysis was performed to determine the reaction specificity. 

For correlation, whole microarray sets were re-analysed by normalizing the data according to *ACT1*, using Genespring Software (Agilent Technologies, Santa Clara, CA, USA) and logarithmic fold-change values were used. For *rho* and *p*-values calculation, Kendall and Spearman tests [37,38] were performed by using R software [39].

### 2.6. Whole Genome Re-Sequencing

Whole genome re-sequencing experiments were performed at Genext Biotechnology and Laboratory Services Ltd., as described previously [26]. The data of this work have been deposited in the NCBI Sequence Read Archive (SRA) under BioProject PRJNA575869.

## 3. Results

### 3.1. Iron Resistance and Cross-Resistance of the Evolved Mutants to Other Transition Metals

#### 3.1.1. Selection of the Evolved Mutants from the Final Population and Their Metal-Stress Resistance Determination Using the MPN Method

The evolutionary engineering strategy consisted of successive batch selection in the presence of gradually increased iron stress levels, starting with 5 mM FeCl_2_ in the first passage, up to 30 mM FeCl_2_ at the 15th passage. As the survival rate of the 15th passage was as low as 0.15, the selection experiments were ended at this final population and the final population was plated on YMM-agar plates. Four individual colonies were randomly picked from the plates, upon incubation at 30 °C for 48 h. The selected mutants called *M3FE*, *M5FE*, *M7FE* and *M8FE* and the final population (FP) were cultivated in YMM containing 2 mM CrCl_3_, 1 mM MnCl_2_, 10 mM FeCl_2_, 35 mM(NH_4_)_2_Fe(SO_4_)_2_, 1 mM CoCl_2_, 0.2 mM NiCl_2_, 0.075 mM CuCl_2_, 5 mM ZnCl_2_ stress factors and the viable cell numbers were determined using the MPN method. As we had also used chloride salts of other metals in our previous studies on evolutionary engineering of cobalt- [21] and nickel-resistant [23] *S. cerevisiae*, we have also used in this study FeCl_2_ during selection experiments. However, as (NH_4_)_2_Fe(SO_4_)_2_ usually had a higher solubility than FeCl_2_, we also used it in the experiments of the present study. 

**Figure 1:**
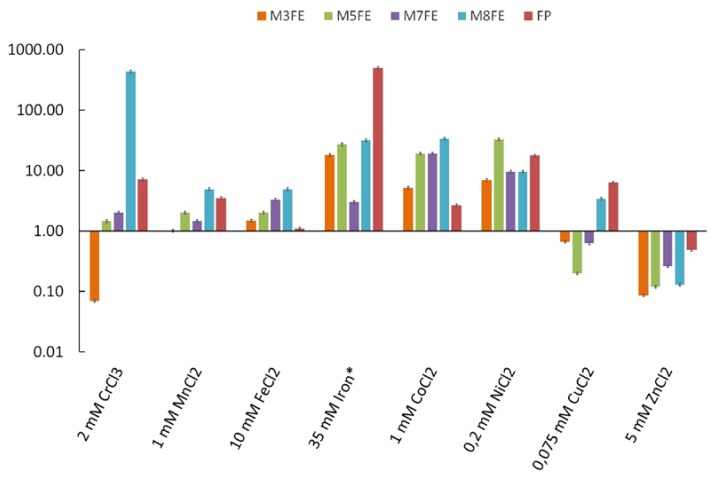
Survival rates as folds of the reference strain (*905*) of the mutants (*M3FE*, *M5FE*, *M7FE*, *M8FE*) and the final population (FP), represented in log-scale. The results were obtained using the MPN analysis under various metal stress conditions. Iron* indicates (NH_4_)_2_Fe(SO_4_)_2_.

Survival rates as fold of the reference strain (*905*) are indicated in Figure 1. According to that, all selected mutants were resistant to 10 mM FeCl_2_ and 35 mM (NH_4_)_2_Fe(SO_4_)_2_, as expected. Additionally, all mutants and FP showed cross-resistance to 1 mM CoCl_2_ and 0.2 mM NiCl_2_ stress and most of the mutants were cross-resistant to 2 mM CrCl_3_ and 1 mM MnCl_2_ stress. On the other hand, all mutants showed significant sensitivity to 5 mM ZnCl_2_ stress. Based on all these results, as the mutant with the highest resistance level in most of the stresses tested, *M8FE* was chosen for further detailed analyses.

#### 3.1.2. Determination of Cross-Resistance to Metal and Non-Metal Stresses by Spot Assay

Spot assays were performed for *905* and *M8FE* under various stress conditions. In line with the MPN results, *M8FE* was resistant to 25 mM FeCl_2_ and 50 mM (NH_4_)_2_Fe(SO_4_)_2_ stresses, compared to *905*. As reported previously [9], iron remains bioavailable, even if it precipitates in solid media. Thus, the inhibitory effects of iron on solid media are more pronounced or reliable, compared to in liquid media. The high concentrations of FeCl_2_ (25 mM) and (NH_3_)_2_SO_4_ (35 mM) used in solid media indicate a significant iron resistance level for *M8FE*.

**Figure 2:**
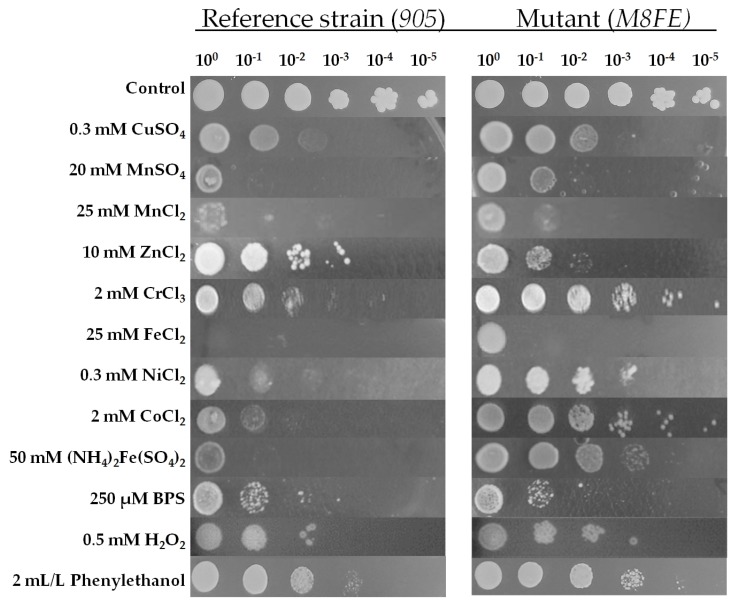
Spot assay profiles of *905* and *M8FE* under a variety of stress conditions. Dilutions are shown from 10^0^ to 10^−5^, from left to right.

*M8FE* also showed significant cross-resistance to 2 mM CrCl_3_, 0.3 mM NiCl_2_, 2 mM CoCl_2_ and 2 mL/L phenylethanol. It was sensitive to 10 mM ZnCl_2_, compared to *905*. *M8FE* did not display any significant resistance or sensitivity to an iron chelator (250 µM BPS) or 0.5 mM H_2_O_2_ as a stress factor (Figure 2).

### 3.2. Physiological Analysis

#### 3.2.1. Iron and Cobalt Contents Determined by Flame–Atomic Absorption Spectrometry (F-AAS) Measurements

To gain insight into the iron resistance mechanism of *M8FE* (e.g., sequestration, avoidance, or both), iron atoms arrested in *M8FE* and *905* were analysed by calculating the “mg Fe atom/g cdw” values obtained from the F-AAS analysis (Table 1). *M8FE* grown in YMM without iron (control group) contained high levels of iron, according to the F-AAS readings, indicating that *M8FE* already arrested iron atoms in or on itself, during the evolutionary engineering selection steps. After subtracting the control condition read values from the stress condition read values of each sample it was observed that the reference strain (*905*) took up more iron than *M8FE*. *M8FE* apparently avoided iron uptake more than *905* did and, even under non-stress conditions, *M8FE* already contained high levels of iron. Similar results were also obtained in the presence of cobalt stress, where the reference strain took up more cobalt than *M8FE* (Supplemental Table S1).

#### 3.2.2. Growth Behaviour and Extracellular Metabolite Profiles (Glucose, Ethanol, Acetate, Glycerol)

**Figure 3:**
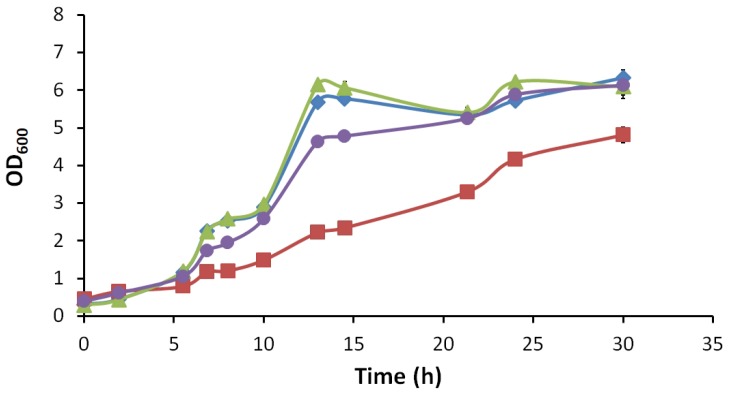
Growth profiles of *905* and *M8FE* in the absence and presence of 15 mM (NH_4_)_2_Fe(SO_4_)_2_ stress. ◆ *905* under control condition, ■ *905* under iron stress condition, ▲ *M8FE* under control condition, ● *M8FE* under iron stress condition.

The growth physiology of *M8FE* and *905* was investigated in batch cultures, in the presence and absence of 15 mM (NH_4_)_2_Fe(SO_4_)_2_ stress. The growth profiles are shown in Figure 3. It was observed that the presence of iron stress in the culture environment significantly reduced the growth of *905*, while *M8FE* was apparently not affected by that iron stress. Additionally, there were no significant differences between the growth behaviours of *905* and *M8FE* under control conditions, implying that the evolved strain does not have any growth deficiency. The maximum specific growth rates (*µ*_max_) of the reference strain and *M8FE* under control conditions and *M8FE* under iron stress condition were all about 0.2 h^−1^. However, the *µ*_max_ of the reference strain under iron stress condition was about 0.1 h^−1^, indicating the strong inhibition of the reference strain by the iron stress.

**Figure 4:**
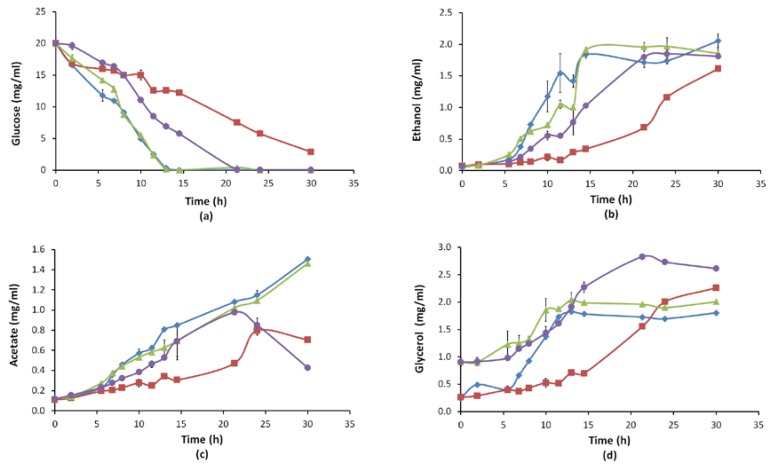
Average glucose (**a**), ethanol (**b**), acetate (**c**) and glycerol (**d**) concentrations (mg/mL) of *905* and *M8FE* in the absence and presence of iron stress (◆ *905* under control condition, ■ *905* under iron stress condition, ▲ *M8FE* under control condition, ● *M8FE* under iron stress condition).

In line with the growth profiles, glucose consumption, ethanol, acetate and glycerol production profiles were also obtained (Figure 4).

Interestingly, *M8FE* grown in the presence of iron stress produced significantly higher amounts of glycerol than all other cultures. Additionally, even in the absence of iron stress; *M8FE* still produced more glycerol than *905* (Figure 4). It was also observed that the presence of iron stress generally reduced the acetate levels, but increased the final glycerol levels of both *905* and *M8FE*.

#### 3.2.3. Reserve Carbohydrate (Trehalose, Glycogen) Profiles

**Figure 5:**
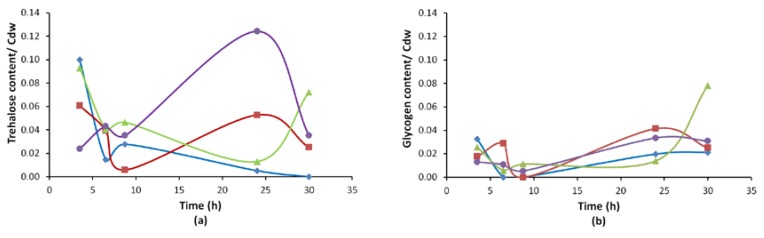
Trehalose (**a**) and glycogen (**b**) contents of *M8FE* and *905* (mg/mg) under control and iron stress conditions (◆ *905* under control condition, ■ *905* under iron stress condition, ▲ *M8FE* under control condition, ● *M8FE* under iron stress condition).

It is known that microorganisms produce reserve carbohydrates like trehalose and glycogen under stress conditions. Additionally, trehalose is also known to play a role in stress protection [40]. The relationship between iron stress and trehalose/glycogen reserves was investigated by enzymatic assays. Trehalose and glycogen contents per cell dry weights are shown in Figure 5.

It was observed that the trehalose content of *M8FE* grown in the presence of iron stress was the highest, compared to all other cultures. However, after 25 h of cultivation, *M8FE* started to consume trehalose in the presence of iron stress (Figure 5).

#### 3.2.4. Oxidative Level Determination

##### Intracellular Reactive Oxygen Species (ROS) Amounts Determined by Fluorescent Intensity Measurements

**Figure 6:**
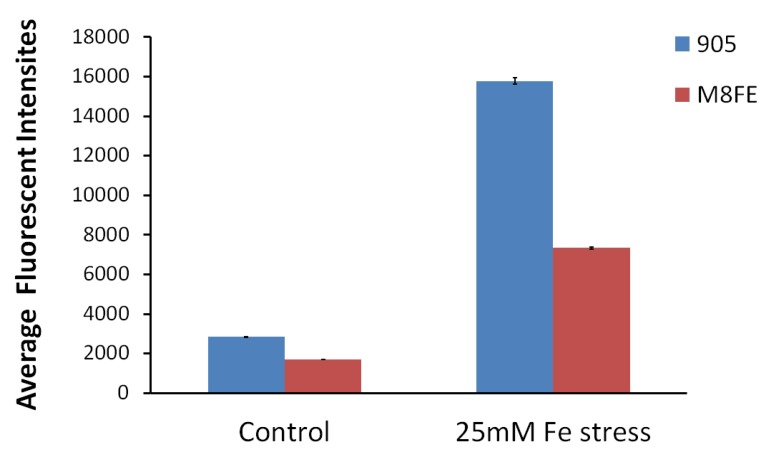
Fluorescent intensity values that indicate the ROS amounts of *M8FE* and *905* under control and iron stress conditions.

Intracellular ROS amounts of *M8FE* and *905* in the presence and absence of iron stress are indicated as fluorescent intensities in Figure 6.

According to dichlorofluorescein (DCF) fluorescence intensities; ROS content of the mutant was 0.6-fold of that of the reference strain under control conditions, while it was only 0.4-fold of it under iron stress conditions. The presence of iron stress significantly increased the intracellular ROS amounts in both *M8FE* and *905*. However, both in the presence and absence of the iron stress, *M8FE* had lower ROS amounts than *905*, implying that the mutant was able to cope with oxidative stress under both conditions.

##### Lipid Peroxidation Assay

Lipid peroxidation assay was employed to determine the oxidative degradation amount of lipids in the cell, an indirect indicator of ROS amounts.

**Figure 7:**
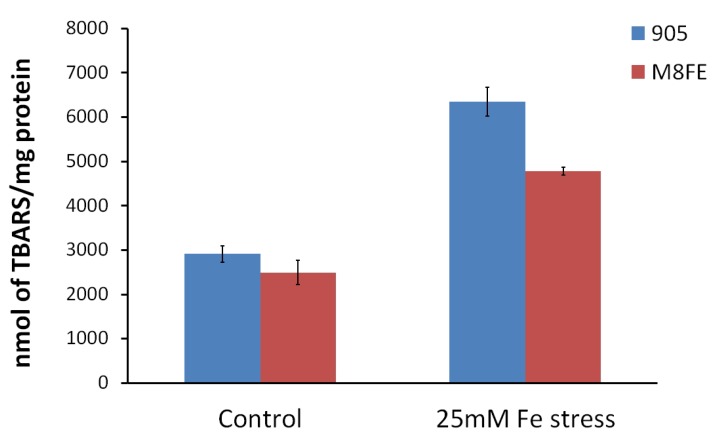
nmol TBARS/mg protein values of *M8FE* and *905*, under control and iron stress conditions.

Absorbance at 535 nm multiplied by the molar extinction coefficient of malondialdehyde (MDA)-thiobarbituric chromophore (1.56 × 10^5^ M^−1^ cm^−1^) indicated nmols of thiobarbituric acid reactive substances (TBARS)/mg protein (Figure 7). It was found that the mutant strain had significantly lower TBARS levels than the reference strain, in the presence of iron stress.

TBARS levels were higher under iron stress conditions for both strains and 0.75-fold of the reference strain in the iron-resistant mutant. According to both oxidative level determination experiments, *M8FE* can apparently cope with oxidative stress better than *905*. Thus, it can be expected that the mutant reduces the high intracellular oxidative levels by producing antioxidant elements.

#### 3.2.5. Lyticase Susceptibility Assay

**Figure 8:**
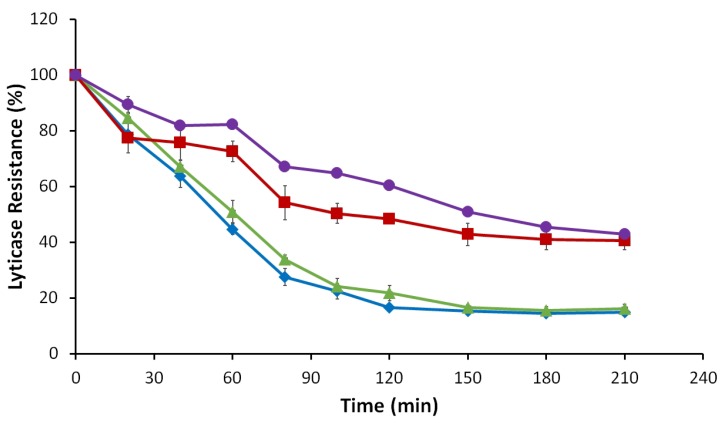
Lyticase susceptibilities of *905* and *M8FE* in the absence and presence of iron stress (◆ *905* under control condition, ■ *905* under iron stress condition, ▲ *M8FE* under control condition, ● *M8FE* under iron stress condition). Lyticase susceptibility was assessed as the percent decrease in lyticase resistance (initial value = 100%).

Lyticase susceptibility assay was performed to evaluate the cell wall integrity of *M8FE* and the reference strain. The results showed that the *M8FE* mutant was significantly more resistant to the lyticase stress than the reference strain, particularly in the presence of 15 mM (NH_4_)_2_(Fe)(SO_4_)_2_ stress in the medium. Under non-stress conditions, both strains had comparable lyticase resistance levels. Additionally, the presence of iron stress in the culture medium increased the lyticase resistance of both strains (Figure 8).

### 3.3. Transcriptomic Analysis Results

#### 3.3.1. One-Color DNA-Microarray Analysis Results

One-color microarray analyses were performed for *M8FE* and *905*, under control and iron stress conditions. Samples for DNA-microarray analyses were withdrawn at an OD_600_ of about 1.0 (5 × 10^7^ cells/mL). The complete microarray data are available at the GEO repository under accession number GSE61317 [41]. Microarray result sets under control conditions were obtained by calculating the fold change of *M8FE* expression under control conditions versus the reference strain (*905*) expression under the same control conditions. It was found that 272 genes were upregulated and 287 genes were downregulated. Similarly, results under stress conditions were obtained by calculating the fold change of an *M8FE* expression under stress conditions versus the *905* expression under stress conditions. It was found that under stress conditions, only seven genes were upregulated and 31 genes were downregulated (Table 2). It was observed that the stress treatment decreased the number of differentially expressed genes in *M8FE*, compared to *905*.

In addition to these two different sets of gene expression analysis, other combinations of gene expression analysis were also performed, such as; a fold change of *905* under stress conditions versus that of *905* under control conditions (Table 2). Finally, two analysis sets that involve the comparison of *M8FE* and *905* under control and iron stress conditions are discussed in this paper.

**Figure 9:**
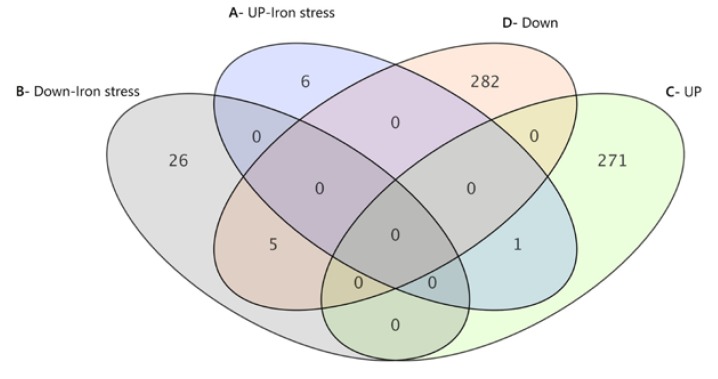
Venn diagrams of common and non-common gene lists; A = {UP-FC ([*M8FE*] vs. [*905*] under iron stress}, B = {DOWN-FC ([*M8FE*] vs. [*905*] under iron stress}, C = {UP-FC ([*M8FE*] vs. [*905*] under control cond.}, D = {DOWN-FC ([*M8FE*] vs. [*905*] under control cond.}. FC indicates fold change.

Up- and downregulated gene lists obtained from two analysis sets were clustered, the common and non-common gene lists were generated and the numbers of genes in these lists are shown as Venn diagrams (Figure 9) (GeneSpring GX 12.0, Agilent Technologies, Santa Clara, CA, USA).

The only gene that was upregulated in *M8FE* under both iron stress and control conditions was *BNA3* (Figure 9). Under iron stress conditions, *BNA3* was upregulated by 2.8-fold, and under control conditions, it was upregulated by 2.2-fold. This gene is responsible for the biosynthesis of nicotinic acid.

The five genes that were downregulated in *M8FE* under both iron stress condition and control condition (with respect to the reference strain) were; *PHO84*, *SPL2*, *UTP30*, *YIG1* and *YPL245W*. They were downregulated by 25.4-, 14.2-, 3.1-, 2.4- and 2.2-fold under iron stress conditions, and 70.5-, 10.2-, 2.4-, 2.8- and 2.2-fold under control conditions, respectively. It is known that the most downregulated gene of the group, *PHO84*, is a high-affinity inorganic phosphate (Pi) transporter and cells over-expressing *PHO84* accumulate heavy metals (Mn^2+^, Cu^2+^, Co^2+^) [42]. Additionally, another common gene and the second most down-regulated gene of the mentioned group, *SPL2*, acts as a low-affinity phosphate transporter during phosphate limitation by targeting Pho87p to the vacuole. *UTP30* is involved in the production of 18S rRNA. *YIG1* plays a role in anaerobic glycerol production. YPL245W encodes a putative protein of unknown function [43]. As these five genes were downregulated in *M8FE*, compared to *905* under both conditions, their expression is most likely independent of the iron stress in the environment.

##### Transcriptome Profiles in the Absence of Iron Stress

*M8FE* expression levels with fold changes higher than 2, compared to *905* expression levels, were detected, and 272 genes were found to be upregulated in the absence of iron stress. Gene ontology analysis of those genes according to their biological processes have been performed using the Funspec tool [44] and the results are indicated in Table 3. Lists of all up- and down-regulated genes as folds of the reference strain in the absence of iron stress are provided in Supplemental Table S2.

Upregulated genes under control conditions have been clustered in several categories. Most of the genes were clustered in biological process and metabolic process categories. A significant number of them was clustered in glycogen and trehalose biosynthetic process categories and some of them in cellular response to oxidative stress category.

*M8FE* expression levels with fold changes higher than 2, compared to *905* expression levels, were detected, and 287 genes were found to be down-regulated in the absence of iron stress. Gene ontology analysis of those genes were performed according to their biological processes, by using the Funspec tool [44], and the results are shown in Table 3.

According to Funspec analysis results, most of the downregulated genes in *M8FE* were categorized under ribosome biogenesis-related groups. The ribosome biogenesis might be reduced by the mutant, possibly because of the high consumption of the cellular energy and the building blocks. 

**Figure 10:**
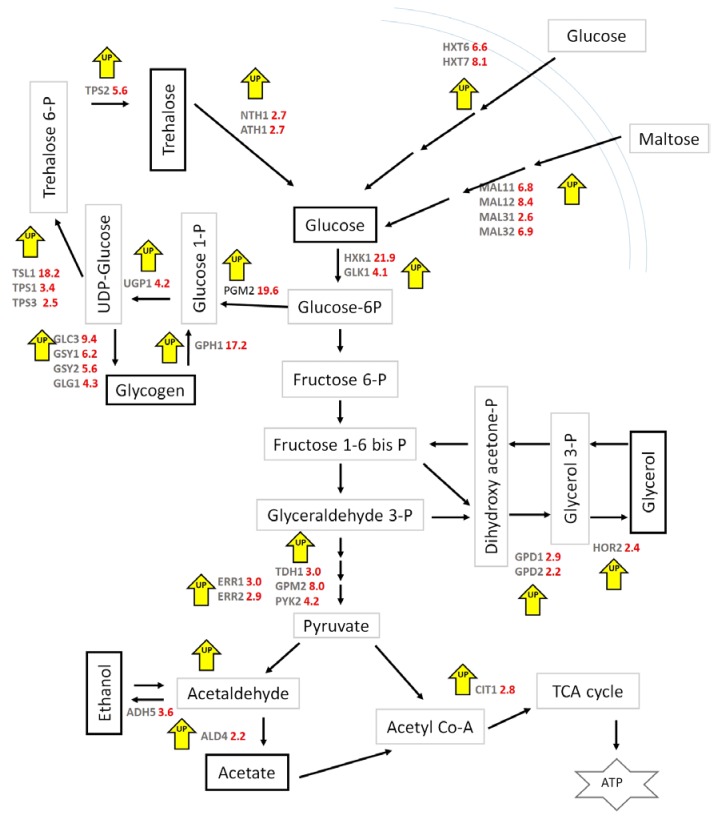
Upregulated genes in *M8FE* and their FC values as folds of *905* on the principal pathway of carbon metabolism (Figure was prepared by using the GeneSpring GX 12.0, Agilent Technologies-Wiki-pathway database [46]).

Figure 10 indicates the differentially-expressed genes in *M8FE* and their fold change values on the central carbon metabolic pathway. Considering glucose as the starting metabolite for metabolic pathways, it is transported by the help of some enzymes and transporters. High-affinity glucose transporter genes *HXT6* and *HXT7* were 6.6- and 8.1-fold upregulated, respectively, in *M8FE* under control conditions. *MAL11*, *MAL12*, *MAL31* and *MAL32*, all responsible for maltose degradation to glucose, were 6.8-, 8.4-, 2.6- and 6.9-fold up-regulated, respectively, in *M8FE* under control conditions. These upregulated genes indicate that glucose can be maintained in the cytosol by the help of those changes in *M8FE*. The transported glucose can then be converted to Glucose-6P by hexokinase encoded by *HXK1* and *GLK1*, which were 21.9- and 4.1-fold up-regulated, respectively, in *M8FE*, under control conditions. However, the levels of *HXK2*, the gene that represses the expression of *HXK1* and *GLK1*, were not different from each other in *M8FE* and the reference strain. Glucose-6P would either then be converted into fructose 6-P or glucose 1-P, by either the glycolytic or the glycogen biosynthetic pathway, respectively.

*PGM2* that encodes phosphoglucomutase either converts glucose-1-phosphate to glucose-6-phosphate or conversely [47], which allows the switch between glycolysis or the glycogen biosynthesis pathway. According to the microarray results, *PGM2* was 19.6-fold upregulated in *M8FE* under control conditions. Thus, the mutant seems to use this switch tightly between glycolysis and glycogen biosynthesis pathways. Glycogen and trehalose biosynthetic pathways seem to be more tightly regulated in *M8FE* than the reference strain, as many of the genes involved in these pathways were upregulated. Regarding the trehalose metabolic pathways, it was observed that not only were the genes related to trehalose biosynthesis, such as *TSL1*, upregulated in *M8FE* (by 18.2-fold of the reference strain), but also the trehalose degradation genes, such as *NTH1* and *ATH1* (both 2.7-fold upregulated in *M8FE)*. These findings suggest that the trehalose metabolism of *M8FE* is active both in biosynthesis and degradation of trehalose, which most likely helps the strain to better adapt to the changes in the environment, including iron stress. Additionally, the genes related to glycerol biosynthesis, *GPD1*, *GPD2* and *HOR2*, were also upregulated, supporting the glycerol production capacity of *M8FE* (Figure 10). 

All genes indicated in Figure 10 were upregulated. Interestingly, there were no downregulated genes on any pathways which were categorized according to the Wiki-pathway database.

###### Transcriptome Profiles in the Presence of Iron Stress

Expression levels of *M8FE* with fold changes higher than 2, compared to *905* expression levels, were detected and seven genes were found to be upregulated. These genes were *JLP1, YKL118W, FMO1, BNA3, AGP3, YML101C-A* and *LMO1* and they were upregulated by 4.50-, 3.66-, *1*3.26-, *3*2.81-, 32.6-, 2.45- and 2.29-fold, respectively. According to these analysis results, 31 genes were downregulated. Like the upregulated genes, most of the downregulated genes are not characterized yet and cannot be clustered in a category. Three of the downregulated genes were phosphate-related genes: *PHO84* (a high-affinity inorganic phosphate (Pi) transporter), *SPL2* (a down-regulator of low-affinity phosphate transport during phosphate limitation), and *PHO92* (a posttranscriptional regulator of phosphate metabolism) were found to be downregulated by 25.49-, 14.24- and 12.85-fold, respectively (Supplemental Table S3).

#### 3.3.2. Validation of Microarray Results Using qRT-PCR

Selected genes with significantly high levels of up- or downregulation in *M8FE* (*HXK1*, *CTT1*, *HSP26*, *STR3*, *HSP104*, *HSP12* and *PHO84)* were used for the validation of the microarray results by qRT-PCR. According to Spearman’s rank correlation test; calculated *p* and *rho* values between qRT-PCR and microarray sets were 0.00007 and 0.86424, respectively [37,38]. The qRT-PCR results were found to be in line with the microarray results, as indicated in Table 4.

### 3.4. Mutations in M8FE Identified by Whole Genome Re-Sequencing Analysis

The whole genomes of the mutant strains *M8FE* and the reference strain *905* were re-sequenced and 36 mutations were identified in *M8FE*, compared to 905. All mutations were found to be missense mutations, intragenic and transition substitutions, as a result of EMS mutagenesis [28] and were distributed among all chromosomes. 

Five genes (*SFI1*, *UTH1*, *EGT2, NOC3* and *STU1)* with missense mutations have biological functions related to cell division activity (Table 5). *SNF6* and *YRM1*, two transcription factors with missense mutations in *M8FE*, are involved in chromatin remodelling and multidrug resistance, respectively [43]. 

The *STT4* and *VPS34* gene products show 26% sequence identity and both play an essential role in phosphatidylinositol-mediated signalling and both were found to have missense mutations in *M8FE* (Table 5). The *STT4* gene product also showed 27% sequence identity to the catalytic subunit of mammalian phosphatidylinositol (PI) 3-kinase [48].

Two genes, *SSN2* and *NUP2*, with missense mutations in *M8FE*, are involved in the nuclear-transcribed mRNA catabolic process and one gene, *ATX2*, is related to metal homeostasis (Table 5).

## 4. Discussion

In this study, we have obtained an iron-resistant *S. cerevisiae* mutant, *M8FE*, which showed cross-resistance to other metals, such as chromium, nickel and cobalt. To gain insight into the cross-resistances of *M8FE* to the transition metals, it is necessary to know about the Earth’s crust and habitat. Iron in the Earth’s crust is most often found in the form of oxides, such as Fe_2_O_3_ hematite, Fe_3_O_4_ magnetite, Fe_2_O_3_.H_2_O geothite, Fe_2_O_3_ maghemite and Fe_2_O_3_.H_2_O lepidocrocite [4]. It can also be found in the form of minerals that have been categorized into several groups, such as pyrite, smaltite and chloanthite. Among these groups, iron-containing minerals generally co-exist with some other metals, predominantly Co and Ni. This is because nickel and cobalt ions combine with iron and manganese hydroxides favourably [49]. However, in other cases, iron can also co-exist with Cu (chalcopyrite group), Pb (henryite), Zn (marmatite group) and Au (schirmerite group) [50]. Additionally, the main mineral source of chromium is the chromate “FeCr_2_O_4_”.

It is also known that cobalt stress induces iron uptake genes in *S. cerevisiae*, as observed in the case of iron starvation [51]. The resulting cobalt resistance found in the iron-resistant mutants *M3FE*, *M5FE*, *M7FE* and *M8FE* was, therefore, not surprising. In a previous study of our group, a cobalt-resistant *S. cerevisiae* mutant obtained by evolutionary engineering was also found to be cross-resistant to several transition metals, such as iron, nickel, zinc and manganese, but not to copper and chromium ions [52]. Similarly, a nickel-resistant *S. cerevisiae* mutant obtained by evolutionary engineering was also cross-resistant to iron, cobalt, zinc and manganese [23].

It has been demonstrated in several studies that pre-conditioning enables cells to be more resistant to later lethal stresses. Sub-lethal doses of stress applications lead to protection and also cross-resistance to different stresses. Mild stress levels induce cells to be adapted so that cells enter an apoptotic-resistant state and increase resistance to stronger stress (a form of hormesis) [53]. Although the gained cross-resistance of *M8FE* to some transition metals like cobalt, chromium and nickel may seem like a pre-conditioning effect, it is important to note that the systematic evolutionary selection strategy with prolonged exposure to iron stress during many successive batch cultivations can be expected to yield a stronger and permanent cross-resistance against other stress types, for which the yeast cells may have similar resistance mechanisms or common molecular factors for resistance, than a simple pre-conditioning in a single batch cultivation.

Our microarray results revealed that *PHO84* was the most downregulated gene in *M8FE* under both iron stress and control conditions. To our knowledge, there are not many reports in the literature that directly connect *PHO84* to iron regulation: It has been reported previously that the *PHO84* overexpression induced heavy metal (Mn^2+^, Cu^2+^, Co^2+^ and Zn^2+^) accumulation in *S. cerevisiae* cells [42]. Additionally, when “*ΔPHO84 S. cerevisiae*” cells were challenged with high concentrations of zinc, cobalt and copper, they survived by preventing metal accumulation in the cell [54]. It has also been previously shown that high levels of cytosolic and non-vacuolar phosphate in *pho80* mutants cause an increase in sodium and calcium levels and sensitivity to manganese, cobalt, zinc and copper. *pho80* mutants cannot sense phosphate, thus phosphate uptake, storage and metabolism are disrupted. This also leads to iron starvation response in *pho80* mutants, where the transcription factor Aft1p that responds to iron starvation becomes activated and the iron transport genes are upregulated in cells that contain high levels of phosphate. *PHO84* transcript levels were also found to be increased by six-fold in *pho80* mutants [55]. Our current study suggests that the mutant’s iron resistance and even its cross-resistance to other transition metals might be related to the down-regulation of *PHO84,* according to the close connection between intracellular phosphate and iron homeostasis. Pho84p functions as an environmental P_i_ level sensor and it is the main permease responsible for the uptake of phosphate into the cell. Pho84p also mediates rapid activation of the PKA (protein kinase A) pathway [56] similar to other transceptors that function both as a transporter and receptor. According to a recent study, the high-affinity iron transporter Ftr1p and high-affinity zinc transporter Zrt1p are also transceptors for the micronutrients iron and zinc in *S. cerevisiae* [57]. However, in *M8FE*, no gene expression changes were detected in *ZRT1,* while *FTR1* was downregulated 2.16 times, as folds of the reference strain.

Jennings (1993) suggested that organisms evolved two major strategies to cope with the metal stress: “avoidance” and “sequestration” [16]. According to F-AAS results, it was found that *M8FE* had high reserves of iron in or on itself (most likely remaining from the evolutionary engineering selection experiment) under control conditions. According to those findings, it was estimated that *M8FE* used the sequestration mechanism to survive most probably by compartmentalization of its iron ions into vacuoles, as the *RTN2* gene responsible for vesicle formation was upregulated in *M8FE* by 11.2-fold, in the absence of iron stress. Rtn2p is a reticulon protein member of the RTNLA (reticulon-like A) subfamily and is responsible for vesicle formation and membrane morphogenesis [58]. The upregulation of the *RTN2* gene is most likely associated with the excess iron storage of *M8FE* in its vacuoles. It was also observed that, under iron stress conditions, *M8FE* prevented iron uptake more than the reference strain, indicating that *M8FE* might also be using the avoidance mechanism to survive under iron stress. Alternatively, *M8FE* might simply avoid further uptake of iron, as it is already loaded with iron under control conditions. Further studies would, thus, be necessary to clarify that.

According to the microarray results, many genes involved in oxidative stress response were upregulated in *M8FE* under control conditions. As the iron content of the mutant was also very high, this would lead to oxidative stress or damage in the cell, as reported previously [59,60]. However, intracellular ROS amounts of the mutant were lower than those of the reference strain, implying that the upregulated oxidative stress-related genes in *M8FE* most likely help reduce the intracellular oxidative levels. One of these upregulated genes, for example, is *TSA2*. Tsa2p cooperates with Tsa1p to remove reactive oxygen species. Tsa1p levels were shown to be highly correlated with iron toxicity levels in cells [61].

Based on our whole genome re-sequencing results, one of the reasons the iron stress-resistant mutant survived during the evolutionary selection strategy might be the missense mutations identified in *ATX2*, *NUP2* and *UTH1* genes, due to their close association with oxidative stress. It has been previously shown that the overexpression of the *ATX2* gene causes cells to accumulate increased levels of manganese and suppresses oxygen toxicity in sod1Δ mutants. Copper/zinc-dependent superoxide dismutase (Sod1p) plays a major role in the detoxification of oxygen free radicals, contributing to coping with the oxidative damage. Atx2p reverses oxidative damage of *SOD1* deficiency. Atx2p localizes to the membrane of the Golgi-like vesicles of *S. cerevisiae* where manganese ions accumulate to maintain manganese homeostasis and trafficking. Deletion of the *ATX2* gene decreases the available intracellular manganese levels. The ability of *M8FE* to cope with oxidative stress might be related to Atx2p. The phenotypic characteristics of *M8FE*, like high level of iron accumulation and cross-resistance to other transition metals, may indicate that *ATX2* also functions in metal homeostasis [62].

Previously, it has been shown that a *NUP2* deleted mutant of *S. cerevisiae* showed sensitivity to sodium arsenite (NaAsO_2_) which generates oxidative stress in cells [63]. According to a global analysis of quantitative sensitivity profile of *S. cerevisiae* with homozygous diploid deletion of *NUP2* under batch cultivation in continuous presence of 3 mM hydrogen peroxide, data were hierarchically clustered within the group of increased oxidative stress resistance function [64].

It has been shown that *uth1* ρ0 petite mutants have resistance to peroxides and sensitivity to superoxide, and the same effect has been observed in grande backgrounds. Additionally, multicopy plasmids showed increased resistance to superoxide and increased sensitivity to peroxides on wild-type cells. In aqueous, neutral pH solutions, O_2_^–^ can behave as a reductant, and H_2_O_2_ as an oxidant for oxidized transition metal complexes of Fe^3+^ and Cu^2+^ so that these opposite phenotypes can be seen. The opposite effects of O_2_^−^ and H_2_O_2_ on *UTH1* may imply that Uth1p may be an Fe-binding protein and playing a role in iron homeostasis. It has been speculated that Uth1p serves as a molecular switch or sensor in the regulation of the oxidative-stress response [65].

*UTH1* is also known to be associated with cell wall robustness and the mitochondrial protein levels, such as citrate synthase [66]. Our lyticase susceptibility assay results confirmed that the cell wall of *M8FE* was indeed more robust or resistant to degradation by lyticase, particularly in the presence of iron stress. Additionally, *CIT2* gene encoding citrate synthase was also upregulated in *M8FE*. 

Mutations in *VPS34* and *STT4*, two genes encoding phosphatidylinositol kinases, also imply changes in signaling pathways and phosphate metabolism. Additionally, the mutation in *YRM1*, encoding a transcription factor involved in multidrug resistance [43], might be important for the iron resistance, as it is known for another pleiotropic drug resistance regulator, encoded by *PDR1*, where the expression of *PDR1*-regulated genes affects efflux and storage of transition metals [67].

ROS are natural by-products of respiration in mitochondria. When oxidative damage accumulates within mitochondria, autophagy of the organelle occurs selectively. This process, called mitophagy, is conserved from yeast to humans. Mitophagy may lead to several neurodegenerative diseases (e.g., Parkinson’s) and aging [68,69]. It has been previously shown that mammalian mitochondrial rRNAs are degraded in response to oxidative stress [70]. Several mitochondrial degradation-related genes, such as *ATG9*, *ATG1*, *ATG7*, *ATG29* and *ICY2,* were upregulated in *M8FE* in the absence of iron stress. Mitochondria provide cellular energy; however, whenever ATP is produced, intracellular ROS levels increase. To prevent high ROS levels and to keep its metabolic activities at base level in order to save energy, the mutant might be degrading its mitochondria. Another possible reason for the upregulation of mitochondria degradation-related genes can be the missense mutation on *UTH1*. As a mitochondrial outer-membrane protein, Uth1p plays a role in mitophagy. *UTH1*-deleted mutants showed a poorly-efficient mitochondria autophagy ability [68]. The missense mutation on *UTH1* may lead to the upregulation of mitochondria degradation-related genes [65].

Acute iron overload or acute stresses can increase stress responsive intracellular ROS amounts. The intracellular ROS can induce programmed cell death (PCD) [71]. If the stress is high enough, it can lead to necroptosis or even necrosis. However, sub-acute stresses lead to a pre-condition that serves to protect against stress-induced PCD. 

As well as acute stress, chronically stressed cells lead to iron overload, where iron accumulates in vesicles in some specific tissues (siderosis), like the brain, stomach and skin, and causes increased levels of ROS. This iron accumulation in vesicles is observed in many neurodegenerative diseases like Alzheimer’s and Parkinson’s. It is not known if those vesicles serve in a protective or a cell-damaging role [9].

The evolutionary engineering selection strategy that is used in this study may serve both as a sub-acute and sub-chronic stress selection strategy. The transcriptomic data of *M8FE* versus *905* under control conditions indicate that the mutant has already been exposed to iron stress during selection or, due to its high iron content, it is still under sub-acute stress levels regarding up-regulation of the stress responsive genes that generate protection against stress-induced PCD.

It is known that the “ribosome biogenesis” process requires high cellular energy [72]. It can therefore be suggested that the mutant prefers to save its energy as trehalose, rather than consuming it in ribosome biogenesis. Ribosome biogenesis is also related to cell division and growth. However, the mutant did not show any growth inhibition under both control and iron stress conditions [72].

Our transcriptomic analysis results revealed that the gene *GPH1*, responsible for glycogen degradation, was also upregulated by 17.2-fold. The glycogen degradation product “Glucose 1-P” can be used as a substrate for trehalose biosynthesis or it can be converted to “Glucose 6-P” by the help of phosphoglucomutase encoded by *PGM2* (19.6-fold upregulated in *M8FE)*. Glucose 6-P would then be either used in glycolysis or glycerol pathways. Glycerol is produced by *S. cerevisiae* to cope with the osmotic stress, to manage cytosolic phosphate levels or to maintain NAD^+^/NADH redox balance [73]. According to our physiological analysis results, high glycerol production of *M8FE* was remarkable, particularly under the 15 mM (NH_4_)_2_Fe(SO_4_)_2_ stress condition. However, *905*, under the same stress conditions, did not produce as much glycerol as *M8FE.* Thus, the glycerol production difference is most likely due to the robustness of *M8FE* against the iron stress condition. However, the ethanol production of *M8FE* was not as much as the glycerol production, implying that the robust *M8FE* under iron stress conditions possibly shifted the ethanol production pathway to the glycerol production pathway.

The genes *GPD1* and *GPD2*, which are responsible for the synthesis of glycerol 3-phophate dehydrogenase, were also upregulated in *M8FE* under control conditions. These results also support the increased glycerol biosynthesis observed in *M8FE*. To our knowledge, there have been no previous reports implying a relationship between metal stress and glycerol production.

According to the physiological analysis results, *M8FE* produced glycerol and trehalose at significantly higher levels than other metabolites. It has been previously reported that trehalose and glycerol synthesis and degradation genes were stress-induced; additionally, trehalose and glycerol were synthesized during the initial stages of stress treatment [74]. Consequently, it can be suggested that the high levels of trehalose and glycerol production and the upregulation of the related genes may help *M8FE* to overcome the high levels of iron stress.

## 5. Conclusions

In this work, the complex molecular mechanisms and physiology of an iron-resistant *S. cerevisiae* mutant were investigated. The results revealed that the iron-resistant mutant had made significant changes in its energetic and storage metabolism, in its oxidative stress response and its iron storage behaviour. Additionally, it became cross-resistant to nickel, cobalt and chromium; and it had significantly lower intracellular ROS levels. The iron-resistant evolved strain seemed to be prepared for the iron stress by upregulating its oxidative stress response genes, downregulating its ribosome biogenesis genes and tightly controlling its reserve carbohydrate biosynthesis and degradation-related genes.

The gene expression data provided by microarray analysis and the whole genome re-sequencing results are generally in line with the growth physiology data of the mutant and the reference strain. Microarray analysis enabled a system-level investigation of the changes in the mutant at the transcriptomic level. Additionally, comparative whole-genome sequencing analysis enabled genome level identification of the mutant in order to endow highlighted genes to other organisms for further studies as evolutionary engineering suggests. The results suggest that the potential role of phosphate transporters and metabolism, cell wall integrity and the multidrug transporters in iron resistance are yet to be investigated in detail.

For a better understanding of the complex molecular mechanism of iron resistance in *S. cerevisiae* further investigations, including functional genomic and proteomic analyses, are necessary. These findings will help elucidate the complex role of iron and other metals in microbial biodiversity, health and nutrition.

## Figures and Tables

**Table 1:** Iron contents of *M8FE* and *905.*

		Read Values from F-AAS Multiplied by the Dilution Factor	Read Values from F-AAS Multiplied by the Dilution Factor–Controls Subtracted (mg/L)	Cell Dry Weight (CDW)/L (mg/L)	Average mg Fe/g cdw Values
***905***	**Control**	0.085		1330	
		0.094		1170	
		0.082		1270	
	**Iron stress**	0.746	0.659	1130	0.5942 ± 0.0103
		0.762	0.675	1120	
		0.750	0.663	1110	
***M8FE***	**Control**	2.909		970	
		2.974		1030	
		2.996		940	
	**Iron stress**	3.395	0.436	900	0.382 ± 0.088
		3.261	0.302	880	
		3.261	0.302	940	

**Table 2:** Up- and downregulated gene numbers of various combinations of gene expression analysis sets according to GeneSpring GX 12.0 (Agilent Technologies) programme. FC indicates fold change.

Analysis Set Name	Number of Upregulated Genes	Number of Downregulated Genes
FC ([*M8FE*] vs. [*905*])	272	287
FC ([*M8FE* stress] vs. [*905*-stress])	7	31
FC ([*905* stress] vs. [*905*])	178	127
FC ([*M8FE* stress] vs. [*M8FE*])	213	208

**Table 3:** Gene ontology analysis results of the upregulated and down-regulated genes in *M8FE*, in the absence of iron stress, according to their biological process, taken out of 2062 categories. For this analysis, Bonferroni correction [45] was performed and the *p*-value cut-off was selected as 0.01 (LSU: large subunit, SSU: small subunit).

Category	Number of Genes from the Input Cluster in the Given Category	Number of Genes Total in the Given Category
**Up-regulated genes**		
Biological process	85	1203
Metabolic process	39	425
Oxidation-reduction process	29	272
Response to stress	26	152
Carbohydrate metabolic process	20	94
Cellular response to oxidative stress	13	67
Glycolysis	9	28
Glycogen biosynthetic process	7	12
Trehalose biosynthetic process	6	7
Maltose metabolic process	6	11
**Down-regulated genes**		
Ribosome biogenesis	109	170
rRNA processing	103	195
Maturation of SSU-rRNA from tricistronic rRNA transcript	33	60
Endonucleolytic cleavage in ITS1 to separate SSU-rRNA from 5.8S rRNA and LSU-rRNA from tricistronic rRNA transcript	27	40
Ribosomal large subunit biogenesis	24	37
Endonucleolytic cleavage to generate mature 5’-end of SSU-rRNA from (SSU-rRNA, 5.8S rrna, LSU-rRNA)	23	29
Endonucleolytic cleavage in 5’-ETS of tricistronic rRNA transcript	22	27
Ribosomal large subunit assembly	19	38
tRNA processing	17	80
Methylation	16	71
Ribosomal small subunit biogenesis	13	24
Maturation of LSU-rRNA from tricistronic rRNA transcript	11	18

**Table 4:** qRT-PCR values of *M8FE* as fold of the reference strain *905* and the corresponding microarray results normalized to *ACT1* as the log fold change of *905* for each selected gene.

Gene Name	qRT-PCR Values of *M8FE*, as Fold of *905*	Microarray (Norm-*ACT1*) Log FC ([*M8FE*] vs. [*905*])
*PHO84*	0.18	−6.17
*HSP12*	3.70	1.95
*HSP104*	4.62	1.96
*STR3*	4.35	2.96
*CTT1*	3.37	3.19
*HSP26*	7.94	3.26
*HXK1*	16.31	4.55

**Table 5:** Selected mutations in *M8FE* compared to *905*.

Gene Name	Genetic Change	Amino Acid Substitution	Description
Cell division			
*SFI1*	c.2624 G > A	S875N	Centrin (Cdc31p)-binding protein required for SPB duplication.
*UTH1*	c.436 A > G	T146A	Mitochondrial inner membrane protein; implicated in cell wall biogenesis, the oxidative stress response, life span during starvation and cell death.
*EGT2*	c.1592 C > T	S531F	Glycosylphosphatidylinositol (GPI)-anchored cell wall endoglucanase; required for proper cell separation.
*NOC3*	c.593 C > T	T198I	Subunit of a nuclear complex with Noc2p and pre-replicative complexes; required for pre-RC formation and maintenance during DNA replication licensing.
*STU1*	c.3416 C > T	T1139I	Microtubule plus-end-tracking non-motor protein; required for the structural integrity of the mitotic spindle.
Phosphatidylinositol-mediated signalling			
*VPS34*	c.2131 G > A	D711N	Phosphatidylinositol (PI) 3-kinase that synthesizes PI-3-phosphate; forms membrane-associated signal transduction complex with Vps15p to regulate protein sorting.
*STT4*	c. 5341 C > T	P1781S	Phosphatidylinositol-4-kinase; required for normal vacuole morphology, cell wall integrity, and actin cytoskeleton organization.
Nuclear-transcribed mRNA catabolic process			
*SSN2*	c.14 C > T	A5V	Subunit of the RNA polymerase II mediator complex; essential for transcriptional regulation.
*NUP2*	c.627 A > G	I209M	Nucleoporin involved in nucleocytoplasmic transport; has a role in chromatin organization.
Transcription factors			
*SNF6*	c.940 G > A	E314K	Subunit of the SWI/SNF chromatin remodelling complex; involved in transcriptional regulation.
*YRM1*	c. 2251 G > A	E751K	Zn(2)-Cys(6) zinc finger transcription factor; activates genes involved in multidrug resistance.
Metal homeostasis			
*ATX2*	c.61 G > A	G21R	Golgi membrane protein involved in manganese homeostasis.

## Supplementary Materials

The following are available online at https://www.mdpi.com/2076-2607/8/1/43/s1. Figure S1: Survival rates of the reference strain *905* and the EMS-mutagenized initial population *906* upon increasing iron stress conditions, Table S1: Cobalt contents of *M8FE* and the reference strain *905* upon 2.5 mM CoCl_2_ pulse stress treatment, as determined by F-AAS (wavelength and slit width 240.7 and 0.2 nm, respectively), Table S2: List of 272 up- and 287 down-regulated genes in *M8FE* in the absence of iron stress, as folds of the reference strain, Table S3: List of 31 down-regulated genes in *M8FE* in the presence of 15 mM (NH_4_)_2_Fe(SO_4_)_2_ as the iron stress factor, as folds of the reference strain. The complete microarray data is available at the GEO repository under the accession number GSE61317 [41]. Whole-genome re-sequencing data have been deposited in the NCBI Sequence Read Archive (SRA) under BioProject PRJNA575869 at https://www.ncbi.nlm.nih.gov/sra/PRJNA575869.
