# Supplementary material for: Evolutionary Engineering of an Iron-Resistant Saccharomyces cerevisiae Mutant and Its Physiological and Molecular Characterization
Source: Microorganisms. 2019 Dec 24;8(1):43. doi: 10.3390/microorganisms8010043 (PMC7023378; doi:10.3390/microorganisms8010043)
Supplement: Supplementary file 1 [file microorganisms-08-00043-s001.pdf]

Table S1. Cobalt contents of *M8FE* and the reference strain *905* upon 2.5 mM $CoCl_2$ pulse stress treatment, as determined by F-AAS (wavelength and slit width 240.7 and 0.2 nm, respectively).

		Read values from F-AAS multiplied by the dilution factor	Read values from F-AAS multiplied by the dilution factor – controls subtracted (mg/L)	CDW/L (mg/L)	Average mg Co/g cdw values
905	Control	0.047		1330	
		0.029		1170	
		0.022		1270	
	Cobalt stress	1.184	1.151	1220	0.9972 ± 0.0643
		1.315	1.282	1200	
		1.345	1.312	1340	
M8FE	Control	0.062		970	
		0.070		1030	
		0.067		940	
	Cobalt stress	0.621	0.555	900	0.5990 ± 0.0154
		0.622	0.556	940	
		0.590	0.524	890	

Table S2. List of 272 up- and 287 down-regulated genes in *M8FE* in the absence of iron stress, as fold of the reference strain.

**Up-regulated genes as fold of the reference strain**		**Down-regulated genes as fold of the reference strain**	
**FC (abs)**	**Gene Symbol**	**FC (abs)**	**Gene Symbol**
21.98	*HXK1*	70.58	*PHO84*
19.61	*PGM2*	10.24	*SPL2*
18.26	*TSL1*	6.60	*GFD2*
17.25	*GPH1*	5.59	*DBP7*
11.47	*EGO4*	5.55	*SPO19*
11.16	*RTN2*	5.17	*BFR2*
9.88	*SOL4*	5.08	*TOD6*
9.80	*RGI1*	4.76	*VTS1*
9.49	*HSP26*	4.61	*REI1*
9.48	*GLC3*	4.56	*SSF1*
9.08	*DCS2*	4.52	*ALB1*
8.94	*GAD1*	4.44	*YGR079W*
8.75	*CTT1*	4.44	*RSA4*
8.45	*MAL12*	4.35	*RRP36*
8.41	*YLR053C*	4.31	*DHR2*
8.16	*HXT7*	4.25	*FAL1*
8.02	*GPM2*	4.24	*DBP2*
7.92	*STR3*	4.22	*ATC1*
7.55	*YGP1*	4.19	*YBR032W*
6.97	*OPT2*	4.14	*YNL162W-A*
6.93	*MAL32*	4.05	*RRN11*
6.90	*MSC1*	3.97	*CYB5*
6.83	*MAL11*	3.96	*YPL044C*
6.78	*YNL194C*	3.95	*HMT1*
6.62	*HXT6*	3.88	*RLP24*
6.26	*YMR206W*	3.88	*UTP25*
6.26	*PHM7*	3.80	*RFU1*
6.20	*GSY1*	3.80	*SOF1*
6.12	*EMI2*	3.76	*UTP14*
6.12	*TMA10*	3.75	*RPC53*
6.11	*HSP82*	3.72	*FCF2*
6.01	*HSP78*	3.72	*HCA4*
6.00	*BDH2*	3.66	*NSR1*
5.90	*YCT1*	3.62	*MAK16*
5.83	*FMP16*	3.59	*CIC1*
5.72	*UIP4*	3.53	*UTP15*
5.66	*TPS2*	3.52	*SPB1*
5.65	*YRO2*	3.52	*IPI3*
5.65	*PIG2*	3.51	*BUD22*
5.60	*GSY2*	3.50	*SHQ1*
5.56	*TFS1*	3.49	*RPA12*

5.36	*SDP1*	3.45	*UTP18*
5.35	*AIM17*	3.44	*LCP5*
5.34	*IGD1*	3.44	*DIP2*
5.10	*HSP30*	3.42	*RAS1*
5.07	*OPT1*	3.40	*YGR283C*
5.06	*YLR149C*	3.37	*UTP6*
5.02	*ALD3*	3.35	*RCL1*
4.94	*BSC5*	3.35	*RIX7*
4.94	*RTC2*	3.35	*YGR035C*
4.91	*YJL163C*	3.34	*NAF1*
4.90	*YGL258W-A*	3.32	*NOG2*
4.85	*GPG1*	3.32	*RRS1*
4.62	*GAC1*	3.30	*IMP3*
4.62	*RTC3*	3.28	*EBP2*
4.57	*TMT1*	3.25	*NRP1*
4.55	*SYM1*	3.22	*PUF6*
4.53	*YNL200C*	3.22	*EFG1*
4.52	*NNR2*	3.21	*YBL081W*
4.51	*OYE3*	3.20	*DBP9*
4.49	*TOS8*	3.18	*CGR1*
4.37	*YNR068C*	3.18	*SDA1*
4.35	*GLG1*	3.17	*ECM1*
4.32	*TPK1*	3.13	*YJR056C*
4.31	*XKS1*	3.12	*DUS1*
4.28	*UGP1*	3.11	*UTP23*
4.24	*PYK2*	3.10	*MAK21*
4.22	*ROQ1*	3.10	*PWP2*
4.18	*GTO1*	3.08	*UTP9*
4.14	*FMP33*	3.08	*UTP13*
4.08	*GLK1*	3.07	*NOP53*
4.07	*YGL117W*	3.07	*SYO1*
4.06	*MET28*	3.07	*AAH1*
4.04	*HSP42*	3.06	*PPR1*
4.03	*GCY1*	3.06	*REX4*
4.00	*SPI1*	3.05	*BUD27*
3.98	*BTN2*	3.04	*RNT1*
3.96	*DAL5*	3.04	*TSR1*
3.96	*HSP104*	3.03	*SAP185*
3.91	*SPR3*	3.03	*SLX9*
3.90	*ISF1*	3.02	*SFL1*
3.89	*SDS24*	3.02	*MRD1*
3.82	*YLR252W*	3.02	*AQR1*
3.80	*APE1*	3.02	*RRT14*
3.79	*YPS5*	3.01	*RRP8*
3.76	*MUP3*	3.00	*HGH1*
3.75	*YNR014W*	3.00	*DBP10*

3.71	*YPL247C*	2.99	*YDR396W*
3.70	*YKL091C*	2.99	*DBP8*
3.63	*ADH5*	2.98	*EFM6*
3.56	*YLR345W*	2.98	*IPI1*
3.52	*OM45*	2.95	*NOC3*
3.48	*RTS3*	2.93	*NOP4*
3.48	*YLR152C*	2.93	*FYV7*
3.47	*HSP12*	2.92	*PHM6*
3.47	*MTH1*	2.92	*RTT10*
3.45	*COX5B*	2.91	*UTP11*
3.45	*TPS1*	2.90	*TRM11*
3.44	*YJR096W*	2.90	*YIG1*
3.41	*SSA1*	2.90	*NOP15*
3.41	*RNY1*	2.89	*TRM2*
3.36	*TSA2*	2.88	*UTP5*
3.32	*YDL124W*	2.86	*MRT4*
3.32	*SSE2*	2.84	*EFG1*
3.28	*TC20762*	2.81	*RRP9*
3.27	*STB2*	2.81	*RIX1*
3.27	*GGA1*	2.81	*YDR526C*
3.21	*GRE3*	2.80	*IZH1*
3.21	*YER188W*	2.79	*EMW1*
3.12	*UBI4*	2.79	*NOG1*
3.10	*EIS1*	2.78	*NOP14*
3.10	*ERR1*	2.78	*UTP7*
3.09	*HSP32*	2.78	*EFM2*
3.07	*YGL262W*	2.78	*NOP8*
3.06	*IRC9*	2.77	*RPL7B*
3.06	*YPT53*	2.76	*NCS2*
3.04	*ROM1*	2.75	*NOP9*
3.04	*XBP1*	2.75	*UTP8*
3.03	*ATG29*	2.73	*BMT6*
3.02	*TDH1*	2.72	*TRZ1*
3.00	*HXT2*	2.72	*ARX1*
2.99	*GPD1*	2.72	*NOC4*
2.98	*FMP23*	2.70	*ECM16*
2.98	*PCL5*	2.70	*UBP10*
2.96	*MEP2*	2.69	*UPS2*
2.96	*YSC84*	2.69	*YPL068C*
2.95	*LEE1*	2.69	*UTR2*
2.95	*ATG1*	2.67	*YGR160W*
2.94	*ERR2*	2.66	*TRF5*
2.90	*TPK2*	2.66	*TSR2*
2.90	*CIT1*	2.65	*SNU13*
2.88	*SAP4*	2.64	*YOR342C*
2.85	*URA10*	2.64	*RRN3*

2.85	*TDA10*	2.64	*YTM1*
2.85	*SNO4*	2.64	*PRM7*
2.84	*PHR1*	2.63	*RRN7*
2.81	*PHM8*	2.62	*KRE33*
2.80	*YMR326C*	2.62	*RPF2*
2.78	*BAP2*	2.62	*TRM8*
2.76	*IKS1*	2.62	*BMT5*
2.75	*SDH6*	2.62	*YLR363W-A*
2.74	*YBR285W*	2.60	*PRP24*
2.74	*CSS1*	2.59	*BMT2*
2.74	*YNL092W*	2.59	*UTP4*
2.74	*DDR48*	2.59	*GRC3*
2.73	*NTH1*	2.59	*FAF1*
2.73	*ATG39*	2.59	*UTP21*
2.72	*YEF1*	2.58	*PRM7*
2.71	*BDH1*	2.57	*YCR016W*
2.70	*USV1*	2.57	*NAN1*
2.70	*ATH1*	2.56	*RPC82*
2.70	*MAL31*	2.55	*SAS10*
2.69	*YOR289W*	2.55	*RSA1*
2.69	*YLR177W*	2.54	*BUD21*
2.69	*RRI2*	2.54	*RRP12*
2.65	*SUL2*	2.54	*OAZ1*
2.64	*BXI1*	2.53	*NOP13*
2.64	*VPS73*	2.53	*DAT1*
2.62	*PTR2*	2.52	*NSA2*
2.62	*YOR186W*	2.52	*YBL028C*
2.61	*GID8*	2.51	*TMA46*
2.61	*ARG1*	2.51	*IFH1*
2.60	*STR2*	2.51	*RPC17*
2.59	*YLR257W*	2.50	*LTV1*
2.58	*TDA1*	2.50	*UTP30*
2.58	*MOH1*	2.49	*PWP1*
2.57	*CYB2*	2.48	*SRO9*
2.56	*VID30*	2.46	*ESF2*
2.56	*YLR031W*	2.45	*PCL1*
2.56	*PIC2*	2.45	*PRP43*
2.55	*UGX2*	2.45	*RPB5*
2.55	*FYV10*	2.44	*ERB1*
2.55	*OPI10*	2.44	*NOC2*
2.53	*YBR056W*	2.42	*URB2*
2.51	*ATG7*	2.42	*FPR4*
2.51	*TPS3*	2.42	*YHL012W*
2.50	*RRT8*	2.41	*RRP14*
2.50	*YAP1801*	2.40	*NOP2*
2.50	*YJL070C*	2.40	*AFG2*

2.49	*YPR127W*	2.40	*UTP22*
2.48	*SAF1*	2.40	*KTI11*
2.46	*PKP2*	2.39	*YDR157W*
2.46	*RTK1*	2.39	*BUD20*
2.45	*HIS4*	2.39	*NOP12*
2.44	*PRX1*	2.38	*FUI1*
2.44	*OM14*	2.38	*MCH5*
2.44	*HOR2*	2.38	*POP3*
2.44	*GND2*	2.37	*YDR514C*
2.44	*RIM8*	2.37	*NOP1*
2.43	*CMG1*	2.36	*HIT1*
2.42	*PCD1*	2.36	*DUS3*
2.41	*ICY2*	2.35	*TGS1*
2.41	*YER121W*	2.35	*YMR310C*
2.40	*GUD1*	2.34	*RRB1*
2.40	*JIP4*	2.33	*AIR1*
2.38	*YMR090W*	2.32	*URB1*
2.37	*ATG42*	2.32	*MTR4*
2.37	*PSK1*	2.32	*RAI1*
2.37	*YBL029C-A*	2.31	*OTU2*
2.36	*GPT2*	2.31	*RRP1*
2.36	*PTK2*	2.30	*INO2*
2.36	*DCS1*	2.30	*GCD10*
2.35	*YMR196W*	2.30	*BUD31*
2.35	*ACH1*	2.30	*YPL245W*
2.35	*RRT1*	2.30	*BUD23*
2.34	*DUR3*	2.30	*ENP1*
2.34	*FRT2*	2.29	*DRS1*
2.33	*RAD59*	2.27	*JJJ3*
2.33	*RIM8*	2.27	*TMA16*
2.32	*PAL2*	2.26	*RIO1*
2.32	*YBR053C*	2.26	*YER187W*
2.31	*MCO32*	2.26	*RPA43*
2.30	*ZTA1*	2.26	*PHO90*
2.30	*YLR030W*	2.26	*LSG1*
2.30	*GSP2*	2.24	*SCC2*
2.29	*MET2*	2.24	*UTP10*
2.29	*RMD5*	2.24	*SUA5*
2.28	*ATG9*	2.24	*DPH1*
2.28	*ORT1*	2.23	*MPP6*
2.26	*BNA3*	2.23	*KRI1*
2.26	*EMP46*	2.23	*NUC1*
2.25	*HSP33*	2.23	*ROK1*
2.25	*YLR446W*	2.22	*IMP4*
2.23	*VHS3*	2.22	*SSF2*
2.23	*MDH2*	2.21	*RCM1*

2.22	*VID28*	2.20	*NCL1*
2.22	*JIP4*	2.20	*RRN6*
2.21	*OXR1*	2.19	*NMD3*
2.20	*GPD2*	2.19	*EMG1*
2.20	*ALD4*	2.19	*BMS1*
2.20	*FMP45*	2.19	*MRX12*
2.20	*RPI1*	2.19	*OPI1*
2.20	*GOR1*	2.19	*RPC19*
2.19	*PRB1*	2.19	*BCD1*
2.19	*RIE1*	2.17	*YOX1*
2.18	*GRX1*	2.17	*VHR2*
2.18	*ATG31*	2.17	*MIG2*
2.18	*MPM1*	2.17	*FTR1*
2.17	*IML2*	2.17	*HO*
2.16	*AGP2*	2.16	*NOP16*
2.15	*CLD1*	2.16	*OGG1*
2.15	*IMA1*	2.16	*AGA1*
2.14	*YMR103C*	2.16	*RPA14*
2.14	*YMR173W-A*	2.15	*YCL021W-A*
2.14	*RIM4*	2.15	*TMA23*
2.13	*UGA2*	2.14	*BCP1*
2.13	*KIN82*	2.13	*NUG1*
2.12	*NQM1*	2.13	*SPB4*
2.10	*VLD1*	2.13	*MOT3*
2.10	*YMR181C*	2.13	*DAS2*
2.09	*SSU1*	2.13	*RPA34*
2.09	*ASI2*	2.12	*MPP10*
2.09	*FMP46*	2.11	*RKI1*
2.09	*ZRG8*	2.11	*RIO2*
2.09	*YAK1*	2.11	*NOB1*
2.09	*YTP1*	2.10	*RRP15*
2.08	*SDT1*	2.09	*THI7*
2.08	*COA4*	2.09	*TRM3*
2.08	*CUR1*	2.08	*DEG1*
2.07	*MYO3*	2.08	*TRM7*
2.07	*FMS1*	2.08	*ELP3*
2.06	*NRG1*	2.07	*RMT2*
2.06	*YGR130C*	2.07	*RBG2*
2.05	*HUL4*	2.07	*RPA49*
2.05	*MCR1*	2.07	*TIF5*
2.03	*VTC5*	2.06	*TRM732*
2.02	*MAL13*	2.06	*NSA1*
2.02	*NIT1*	2.06	*THI80*
2.02	*PDE1*	2.06	*ACL4*
2.01	*YGR237C*	2.06	*SMY2*
2.01	*SOL1*	2.05	*CTP1*

2.01	*YMR262W*	2.05	*IBD2*
-	-	2.05	*NCA3*
-	-	2.04	*NOP19*
-	-	2.04	*NOP10*
-	-	2.04	*FCF1*
-	-	2.04	*RLI1*
-	-	2.04	*BRX1*
-	-	2.03	*SDS23*
-	-	2.02	*MAK5*
-	-	2.02	*RRP17*
-	-	2.01	*KTR2*
-	-	2.01	*TRM1*
-	-	2.01	*DBP3*
-	-	2.00	*SIR2*
-	-	2.00	*TAD2*
-	-	2.00	*HPT1*

Table S3. List of 31 down-regulated genes in *M8FE* in the presence of 15 mM $(NH_4)_2Fe(SO_4)_2$ as the iron stress factor, as fold of the reference strain.

Down-regulated genes as fold of the reference strain in the presence of iron stress	
FC (abs)	Gene Symbol
25.49	*PHO84*
15.47	*ECM11*
14.24	*SPL2*
12.85	*PHO92*
10.73	*Q0182*
9.33	*YLR296W*
7.86	*YNL114C*
6.04	*YBR051W*
5.98	*HES1*
5.41	*YML122C*
5.27	*BAP3*
4.77	*YKL225W*
4.72	*YER091C-A*
4.24	*YNR077C*
3.96	*YER085C*
3.78	*YPL182C*
3.63	*SMK1*
3.63	*COX3*
3.55	*YIL058W*
3.34	*YPR202W*
3.24	*POP6*
3.10	*UTP30*
2.51	*YFL064C*
2.42	*YIG1*
2.34	*ARO9*
2.32	*SPC24*
2.25	*YPL245W*
2.22	*SMD1*
2.20	*YOR300W*
2.14	*RPS27B*
2.04	*YLR462W*

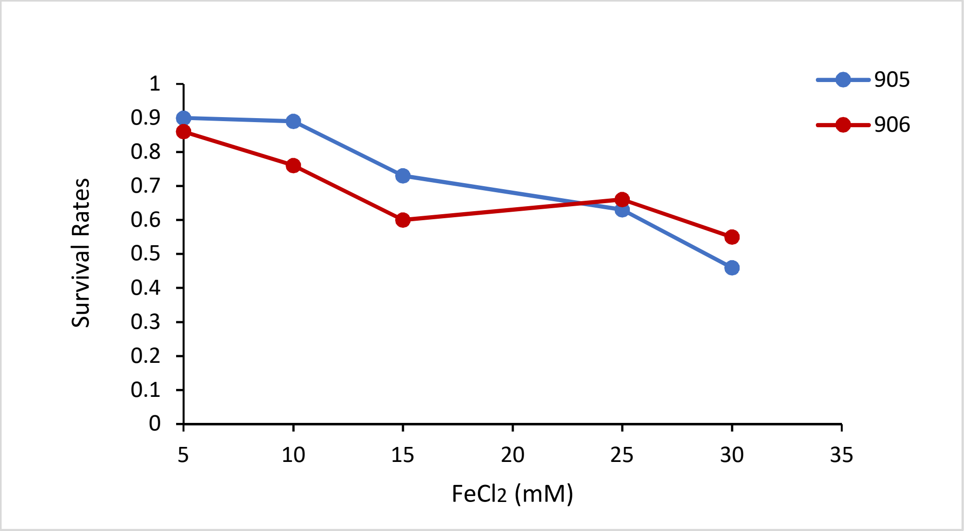

Figure S1. Survival rates of the reference strain *905* and the EMS-mutagenized initial population *906* upon increasing iron stress conditions.
